# Temporal Stability and the Effects of Training on Saccade Latency in “Express Saccade Makers”

**DOI:** 10.1371/journal.pone.0120437

**Published:** 2015-03-20

**Authors:** Paul C. Knox, Felicity D. A. Wolohan

**Affiliations:** Eye and Vision Science, Institute of Ageing and Chronic Disease, University of Liverpool, Liverpool, United Kingdom; Centre de Neuroscience Cognitive, FRANCE

## Abstract

The temporal stability of saccade latency, and the effects of training, particularly in “express saccade makers” (ESMs), has received little attention. ESMs are healthy, naïve, adults, who persist in executing very many low latency “express saccades” (ES; saccades with latency of 80 ms to 130 ms), in conditions designed to suppress such responses. We investigated the stability of ES production (%ES) in 59 ESM and 54 non-ESM participants in overlap tasks. Within a single session, the intraclass correlation coefficient (ICC) for %ES in two runs of 200 trials was 0.97 (*p*<0.001); participants in whom >30% of saccades over the two runs were ES, were classified as ESMs. For 60 participants tested over two sessions 12 weeks apart, and 30 participants tested in three sessions over approximately six months, the ICC for %ES was uniformly high (0.95, p<0.001 and 0.97, p<0.001 respectively) and participants behaved consistently with their initial classification. Fourteen participants (7 ESMs) were then exposed to training consisting of either gap or overlap tasks. Training increased %ES in both groups. However, when tested in overlap tasks, it was not sufficient to transform Normal participants into ESMs. We conclude that the pattern of saccade behaviour exhibited by ESMs constitutes a stable and distinct oculomotor phenotype.

## Introduction

The measurement of saccadic eye movements provides a means of examining both an important aspect of human visuomotor behaviour, and a range of cognitive processes which saccades have been shown to reflect. Beyond description, alterations in specific saccade parameters can often be linked to both specific neural circuitry, and specific aspects of cognition. However, most studies consist of single snapshots of participant performance, usually captured using summary descriptive statistics.

In a number of recent studies we have investigated the saccade behaviour of “express saccade makers” (ESMs). Express saccades (ES) are a neurophysiologically distinct type of saccade. Single-unit recording in non-human primates has demonstrated that they occur when a general reduction in descending inhibition to the superior colliculus allows the visual (target onset) response burst in collicular saccade-related neurons to trigger the brainstem saccade generating network directly [1,2]. This contrasts with the more usual pattern in which the visual burst is followed (later in time) by a second motor burst which triggers brainstem structures. In human behavioural studies, in which this level of neurophysiological information is not available, ES are defined by latency, and less commonly, by the appearance of a distinct early peak in the latency distribution. We have used a latency range of 80ms to 130ms to define ES, a range which is comparable to that used in other studies [3–5]. This compares with a latency of 200ms often quoted as the “normal” latency of human visually guided saccades[6].

As originally described [7,8], ESMs were adult participants, who exhibited extremely high numbers of ES in gap tasks, in which there was a blank period (the gap) between a fixation target being extinguished and the appearance of an eccentric target towards which participants were instructed to execute a saccade. More importantly, this marked overproduction of ES persisted in overlap tasks in which the fixation target remained illuminated when the saccade target appeared. In most participants, the continuing presence of the fixation target inhibits the generation of ES, reducing the proportion in many cases to near zero. In contrast, ESMs persisted in executing a high proportion (>30%) of ES, and their saccade latency distributions often exhibited a pronounced peak around a latency of 100ms. However, it has been suggested that ESMs are likely to be encountered infrequently [9], and this was apparently confirmed by a number of studies with large participant groups which did not report the presence of ESMs [10,11]. Thus it was difficult to study their behaviour in any detail.

In three separate studies we found that ESMs, defined by the proportion of ES executed in *overlap* conditions (>30%), make up a sizable proportion of Chinese participant groups (29% [12]; 22% [13]; 27%[14]), allowing us recruit them in larger numbers than previously and to study them in more detail. We confirmed that the phenomenon was the identical to that described in the earlier literature [12,13], and that the reason for the higher numbers of ESMs among Chinese participants was not due to some cultural influence [14]. We also found that although voluntary saccade performance in ESMs was compromised (we confirmed that they exhibited higher antisaccade error rates than non-ESM participants [13]) this was not due to a general deficit in oculomotor inhibitory control [15].

There is limited evidence in the literature about the stability of express saccade production over time [16,17], and currently no information for ESMs. If it were unstable and on a different day participants identified as ESMs did not reach criterion (or alternatively participants originally identified as non-ESMs did), then the ESM concept would be of reduced value. We would conclude that the production of ES was state dependent rather than a distinct trait. Further, it has been shown that the proportion of ES executed in *gap* conditions can be increased by means of daily training [5,18]. If training could be shown to increase the proportion of ES in *overlap* conditions, then it might be possible to “train” a naïve non-ESM participant to be an ESM. This would suggest that over time some environmental factor, perhaps allied to an underlying sensitivity in particular participants, might lead to the development of an overexpression of ES in overlap conditions. An alternative possibility is that the proportion of ES executed in overlap conditions is stable over time, and remains consistently high in ESMs. Training might not increase ES production in overlap conditions (or at least might not increase it to the extent that non-ESM participants have to be classified as ESMs). This would be compatible with the view that the oculomotor behaviour of ESMs represents a distinct and stable “oculomotor phenotype”. ESMs would therefore be an important group and comparison with non-ESM participants would provide new approaches to investigating both different elements of saccade behaviour [13,15], and aspects of the cognitive processing to which saccades are linked (e.g. different aspects of executive function).

We have therefore conducted two experiments comparing ESM and non-ESM participants. In the first experiment, we examined the stability of the proportion of ES executed in overlap conditions between two blocks within a single session, and then over two or three sessions in large participant groups. In a second experiment, two smaller groups were exposed to training regimes using both gap and overlap tasks to examine the extent to which this affected the proportion of ES executed, particularly in overlap conditions.

## Methods

### Ethical Approval

Experiments were conducted with the approval of the University of Liverpool Ethics Committee and conformed to the standards of the Declaration of Helsinki. Participants were recruited in and around the University of Liverpool; all provided written, informed consent, were paid £5 per testing session and had normal or corrected-to-normal visual acuity.

### Apparatus and stimuli

Horizontal eye movements were recorded binocularly with the same miniaturized head-mounted infrared saccadometer (Ober Consulting Ltd, Poland) used in previous studies[12–14]. This samples infrared reflectance signals at 1KHz, and low-pass filters them at 250 Hz with 12-bit resolution. The device incorporates three forward-facing low-power red lasers projecting red 13 cd/m^2^ spots, to provide fixation and saccade targets; these subtend approximately 0.1°, and are positioned horizontally, centrally and at 10° to left and right of centre. As the lasers move with the head, and the stimuli are therefore head-fixed rather than earth-fixed, we did not stabilize participants’ heads; they sat in a comfortable position approximately 1.5m in front of a near white surface onto which target were projected. Participants were instructed not to move their heads, and were observed throughout data collection to ensure that they did not. Recordings were made with the same level of room illumination in all sessions.

Two types of tasks were run in blocks of 200 trials. In gap tasks, after a randomised fixation period of 1s-2s, the central fixation target was extinguished 200ms prior to the appearance of the saccade target, presented randomly 10° either to the right or left. In overlap tasks, the central fixation target remained illuminated throughout the trial. Again, after the randomised fixation period the saccade target appeared. Regardless of trial type, participants were instructed to saccade to the eccentric target as soon as it appeared, pause, blink and then return their gaze position to the centre in preparation for the next trial.

### Experimental Procedures

Experiment 1. In the first session, 113 healthy, naïve, adult participants completed two blocks of 200 overlap trials. 60 of these participants returned for a second session on average 87±79 days (mean±SD) later and 30 returned for a third session, 94±50 days after their second session, and 178±70 days after their first session. In follow-up sessions, data were available from at least one block of overlap trials. In some of these sessions participants also completed other task types which we have discussed elsewhere.

Experiment 2. In order to investigate whether the proportion of ES could be changed by exposure to practice trials, a smaller group of participants was exposed to a training regime. Fourteen of the above participants were recruited on the basis of their performance before the training baseline measurements, and partly on their availability to commit to the training regime. All fourteen completed the gap training (seven ESMs), and twelve (six ESMs) completed both gap and overlap training. Eight participants completed gap training first and then undertook overlap training. Four participants completed overlap training first. Participants completed the sessions at similar times each day whenever possible. The two training phases were, on average, 11 weeks apart.

**Fig 1:**
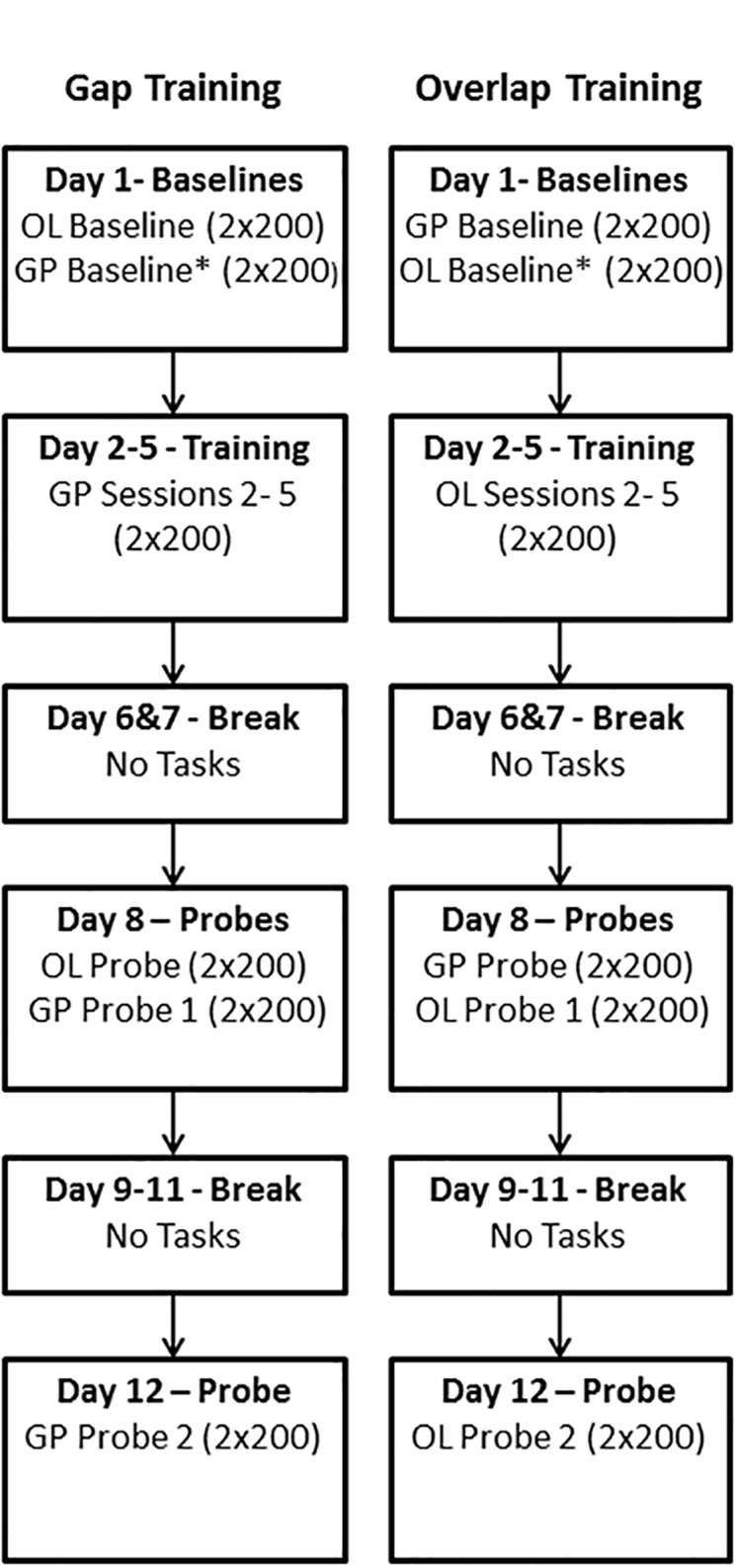
Sequence of Gap and Overlap training regimes. GP: Gap tasks, gap duration = 200ms; OL: Overlap tasks, in which the fixation target remains visible when the saccade target appears to the left or right of fixation. Note the difference in gap and overlap task order in baseline and probe sessions.

For both gap and overlap training the basic regime was identical (Fig. 1). Participants were instructed throughout simply to look at targets as soon as they were aware of them; they were not given specific instructions about speed or accuracy. Training took place over 12 days (Fig. 1). On the first day participants completed 2x200 overlap and 2x200 gap baseline trials with the order depending on which training regime was to follow. For the gap regime, the gap trials were completed after the overlap trials and participants then completed 2x200 gap trials on four consecutive days (training sessions 2–5). After a two day break they returned for their first ‘probe’ session of 2x200 overlap trials (overlap probe) and 2x200 gap trials (gap probe 1). After a further 3 day break they provided 400 gap trials (gap probe 2). For overlap training the sequence was identical except that the order of the baseline and probe tasks was reversed, and probes 1 and 2 consisted of overlap trials (Fig. 1).

### Analysis

Data were stored on the Saccadometer handset and downloaded via an optical/USB link using supplied software (Latency Meter 4.0) before further collation and analysis of saccade latency and amplitude using MS Excel. Statistical analysis was performed using SPSS 16.0. Only saccades with latencies of between 50ms and 500ms were included in the analysis. Median saccade latency and the percentage of ES (saccades with latency in the range of 80 ms to 130 ms; %ES) for each participant were calculated. The intersubject mean (±SD) of individual median saccade latency and the mean (±SD) percentage of ES were used to summarise group performance. We also used average percentage frequency distribution histograms [13,14] to investigate group latency distributions.

Based on the performance on their first test session, in which participants were exposed to a total of 400 overlap trials, those who generated more than 30% of their saccades within the express latency range (80 ms to 130 ms) were classified as “express saccade makers” (ESMs). The remainder of the non-ESM participants will be referred to throughout as “Norms”.

Repeated measures ANOVAs were used to investigate differences between groups (ESM vs Norm; usually as a between-subjects factor as noted in the text) and the influence of various factors on group performance (usually treated as within-subjects factors). Estimates of performance stability over time were obtained by calculating the intraclass correlation coefficient (ICC) for %ES within and between testing sessions. The ICC is an appropriate statistical method in reliability analysis for assessing temporal stability of saccadic measures [19] and has been used in a number of other studies on the temporal stability of saccade task performance [20–22].

## Results

### Experiment 1: Stability within one testing session

**Fig 2:**
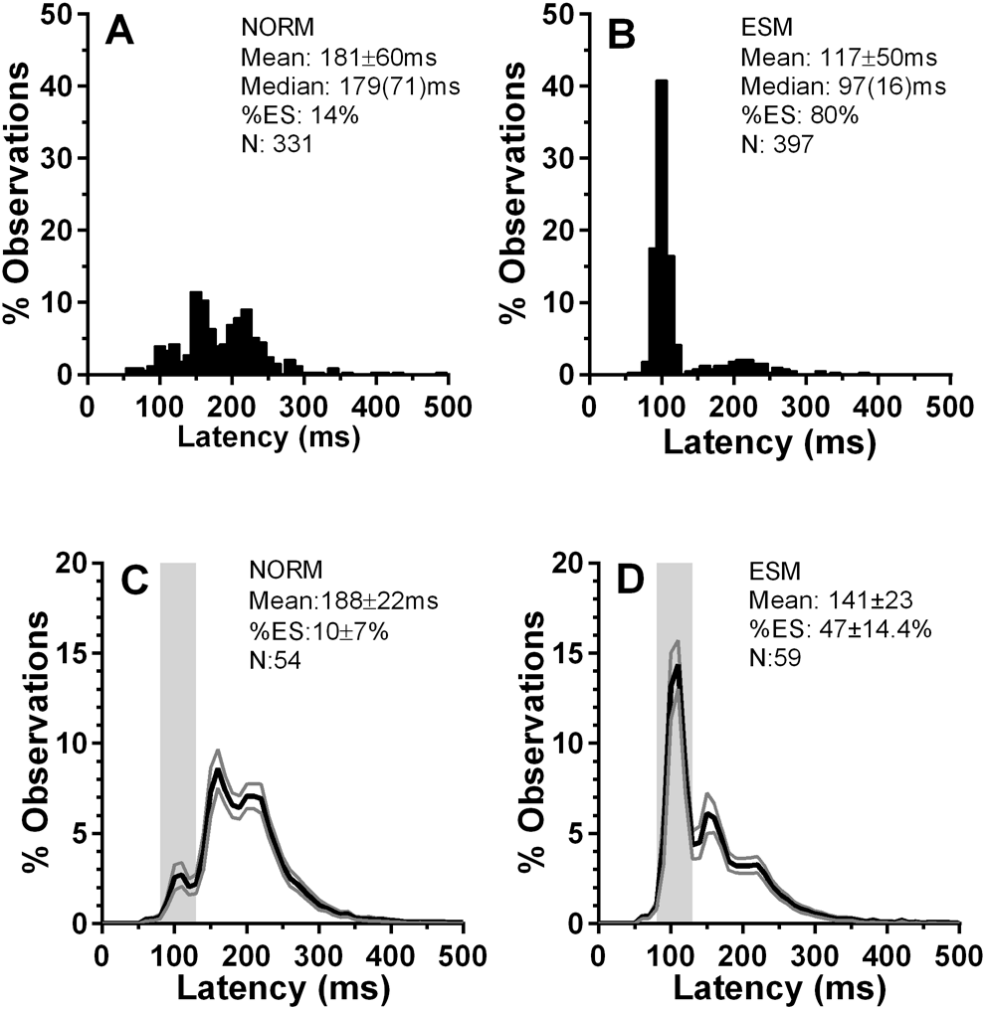
Distribution of saccade latency for the prosaccade overlap task in “express saccade makers” (ESMs) and non-express saccade maker (“NORM”) participants. A,B. Examples of individual distributions with summary parameters (mean±SD; median(IQR)), where N is the total number of observations. C,D. Average distributions for the two participant groups. The values plotted in each 10ms bin are the group mean±95% CI. The solid black line plots the mean, with grey lines showing ±95% CI. The vertical grey region indicates the express saccade latency range (80ms to 130ms). The intersubject mean of individual median saccade latency (±SD) and the intersubject %ES is shown for the two participant groups. N is the number of participants in the group. Note the different y-axis scales between A,B and C,D.

Data were available from a total of 113 participants who attended for at least one testing session in which they were exposed to 400 overlap trials (in two blocks of 200 trials). Of these, 59 generated >30% ES, that is saccades with latency in the express latency range (80 ms–130 ms), and were therefore classified as ESMs (46 Chinese; 13 Caucasian). The remaining 54 non-ESM participants were classified as “Norms” (25 Chinese; 29 Caucasian). Example saccade latency distributions for two individual participants are shown in Fig. 2A and B. For the groups thus defined, average percentage distribution histograms were constructed; each 10 ms histogram bin plots the percentage of observations averaged across the group for that latency bin (with ±95% CI; Fig. 2C,D). As with the individual ESM distribution (Fig. 2B), in the average distribution (Fig. 2D) there was a prominent early peak in the express saccade latency range (centred on 100ms in the individual participant, and 110ms for the group average; bin value 14.4±5.3%; mean±SD). For the Norm group, this peak, while present, was greatly reduced (2.7±2.5%). Group mean percentage of express saccades (%ES) for the ESMs was 47±14.4% compared to 10±7% for the Norms, respectively.

**Fig 3:**
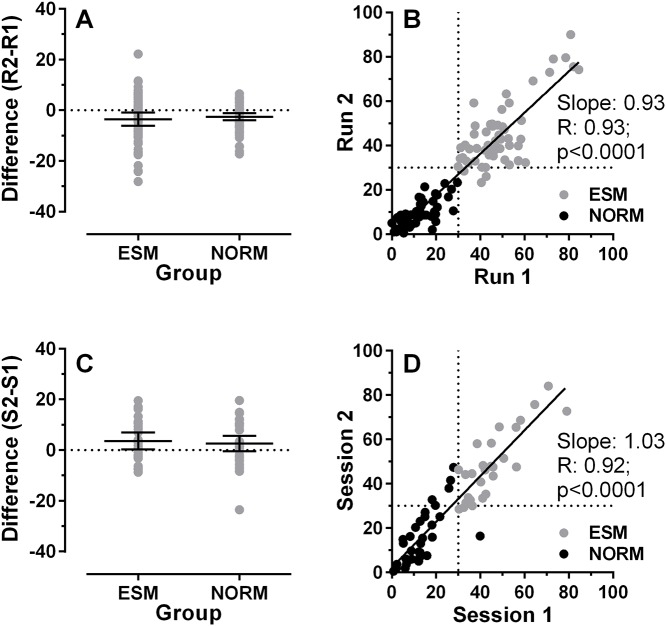
Stability of %ES within sessions (A,B) and between sessions 1 and 2 (C,D). A. Difference in %ES for each participant between two runs of 200 trials within session 1 (calculated as Run 2-Run 1). Group mean±95% CI is shown for ESM and Norm groups. B. Correlation between %ES in Run 1 and Run 2. Least-squares linear regression line is shown with Pearson correlation coefficient and the p value for the correlation. Dotted horizontal and vertical lines show the 30% criterion level for defining a participant as an ESM, based on %ES from the two runs combined. C. Difference in %ES for each participant in Session 1 and Session 2. Other conventions are as in A. D. Correlation between %ES Session 1 and Session 2. Other conventions are as in B.

We compared the performance (%ES) of ESM and Norm groups between the two runs of 200 trials in the first session to assess intrasession stability. For ESMs the mean %ES was 48.7% and 45.2% in the first and second runs, respectively. For the Norms the comparable figures were 11.4% and 8.9%. We examined this further by calculating the difference between runs for each participant (Run 2-Run 1; Fig. 3A), and found that the mean difference was-3.5±10% for ESMs, and -2.5±5.1% for the Norm group (t = -0.67; p = 0.25). As illustrated in Fig. 3B, there was a statistically significant correlation across the whole sample for the %ES in the two runs (ICC = 0.97, *p*<0.001; Fig. 3B also shows the Pearson correlation results). 55/59 ESMs (93%) exhibited >30% ES in both runs. There were 4/59 who dipped below 30% ES on one run (one on Run 1 and three on Run 2).

### Experiment 1: Stability between sessions over time

Of the 60 participants who completed a second test of performance in overlap conditions in a separate session, 28 were classified as ESMs based on their performance in the first session (400 trials), the remaining 32 were Norms. Group mean percentage of express saccades (%ES) for the ESMs was 46.1±14.3% compared to 14.3±10.1% for the Norms, respectively. Additionally, there was a slightly lower percentage of ES in the first session compared to the second which occurred in both groups (overall: 28.7% vs 31.7%; ESMs: 44.8% vs 47.4%, Norms: 12.6% vs 16%). Statistical analysis, using a repeated measures ANOVA with session (1 vs 2) as a within factor and group (ESM vs Norms) as a between factor, confirmed that the percentage ES difference between the groups was significant (F_1,58_ = 117.6, p < 0.001), as was the effect of session (F_1,58_ = 7.6, p = 0.008). Using the same analysis adopted for the intrasessional comparison, we found that the mean difference between sessions was 3.62±8.36% for ESMs and 2.58±8.53% for Norms (Fig. 3C). Again there was a highly statistically significant correlation across all participants for the %ES in the two sessions (Fig. 3D; ICC = 0.95, p<0.001). Only 1/28 ESMs failed to exhibit >30% ES in the second session, and 4/32 participants defined as Norms on the basis of their performance in the first session, exhibited >30% ES in the second session. Therefore 55/60 (92%) participants performed consistently with their classification over the two sessions.

**Fig 4:**
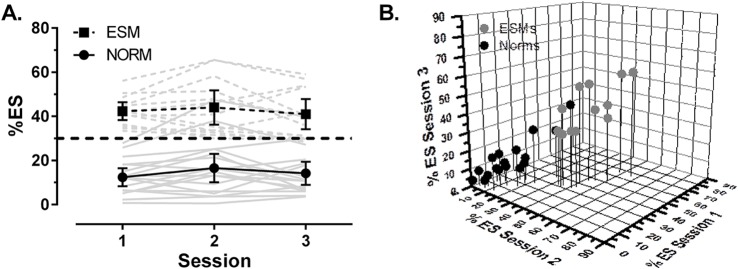
Stability of %ES over three testing sessions. A. Grey lines: individual participant data (broken lines: ESMs; solid lines: Norms). Black lines and points: group data, shown as the mean±95% CI (ESM ■; Norm ●). Black dashed line shows the 30% criterion level. B. 3D correlation plot of %ES over three sessions for ESMs (●) and Norms (●).

Thirty participants provided overlap data on three occasions (13 ESMs, 17 Norms; see Fig. 4). One Norm, defined on the basis of their performance in the first session, exhibited >30% ES in the second and third session and another exhibited >30% ES in the second session only. Thus, 28/30 (93%) participants performed consistently with their classification over the three sessions. Using a repeated measure ANOVA with session (1vs2vs3) as a within factor and group (ESM vs Norm) as a between factor we confirmed that the %ES was significantly different between groups, now across the three sessions (F_1,28_ = 63.8, p < 0.001; ESMs = 42.5±10.4%, Norms 14.4±10.4%). For these results, there was no main effect of session (F_1,30_ = 2.9, p = 0.07; 25.4±16.8% vs 28.5±18.6% vs 25.8±17.1%). The relationship between performance over three sessions is illustrated by means of a 3D plot (Fig. 4B). Again there was a highly statistically significant correlation across all participants for the %ES in the three sessions (ICC = 0.97, p<0.001).

**Fig 5:**
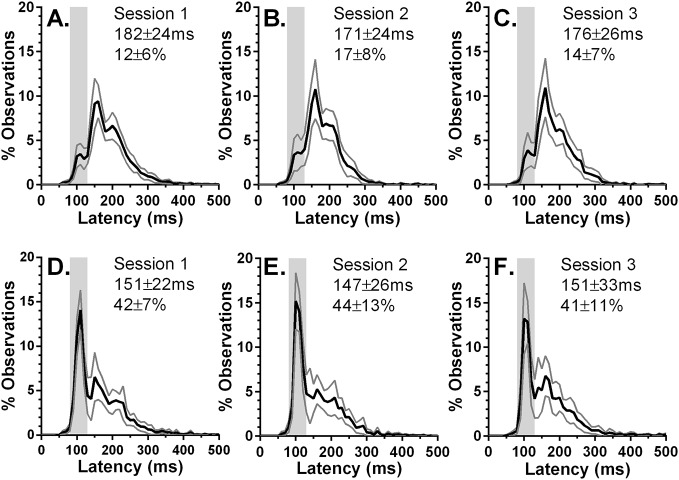
Average (mean±95%CI) percentage latency distribution histograms for the two participant groups. Bin width = 10ms. Intersubject group mean (±SD) latency and intersubject mean %ES are also shown. Grey area indicates the ES latency range (80ms-130ms). A-C: data from Norms; D-F: data from ESMs.

To compare performance across three sessions further, we computed average percentage histograms for both groups and each session (Fig. 5). For each of the ESM distributions (Fig. 5A-C) there was a marked peak in the ES range that was clearly larger than for the Norms (Fig. 5D-F). The clearest peak in the Norm distributions occurred much later in the distribution. This “fast-regular” (FR) peak was much higher than observed in the ESM distributions. However, for both groups the shape of the distributions, and the magnitude and position of peaks, were consistent across the three sessions.

For each session we took the peak and two neighbouring bin values for the ES peak (90ms, 100ms and 110ms for sessions 1 to 3 respectively), and the FR peak (150ms, 160ms and 170ms) for both ESMs and Norms. We then performed a repeated measures ANOVA treating peak (ES v FR), bin (1,2,3) and session (1,2,3) as within subjects and group (ESM vs Norms) as a between subjects factor. Bin (F_2,56_ = 25.5, p<0.001) generated a significant result as did the bin x peak (F_2,56_ = 36.9.4, p<0.001) and bin x group (F_2,56_ = 9.1, p<0.001) interactions. As expected, group was also statistically significant (F_2,28_ = 9.4, p = 0.005) and importantly also the peak x group interaction (F_2,28_ = 37.7, p<0.001). Post hoc tests for group demonstrated that the ES peak was significantly larger in the ESMs than in the Norms (ESMs = 10.1%, Norms = 2.6%; p<0.001), and the FR peak was significantly larger for the Norms (ESMs = 5.5%, Norms = 9.2%; p = 0.009). There was no effect of, nor interactions with, session.

### Experiment 2: Effects of gap training

**Fig 6:**
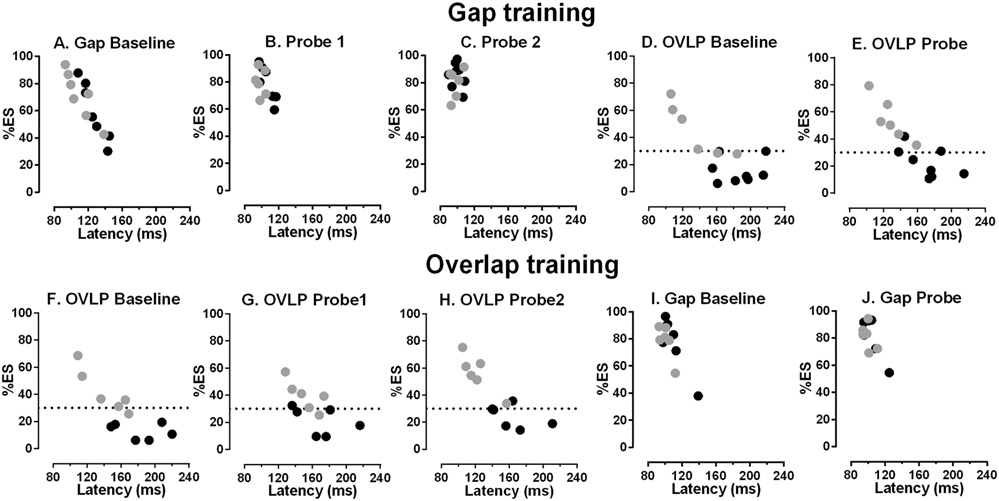
Effect of gap (A-E) and overlap (F-J) training (see Fig. 1 for details of the training regimes). Percentage of express saccades (%ES) is plotted against median saccade latency for ESM (●) and Norm (•) individual participants. For details of the relative timing of different tests in the two training regimes refer to Fig. 1. In plots showing overlap performance during gap (D,E) and overlap training (F-H), the horizontal dashed line is plotted at 30%.

**Fig 7:**
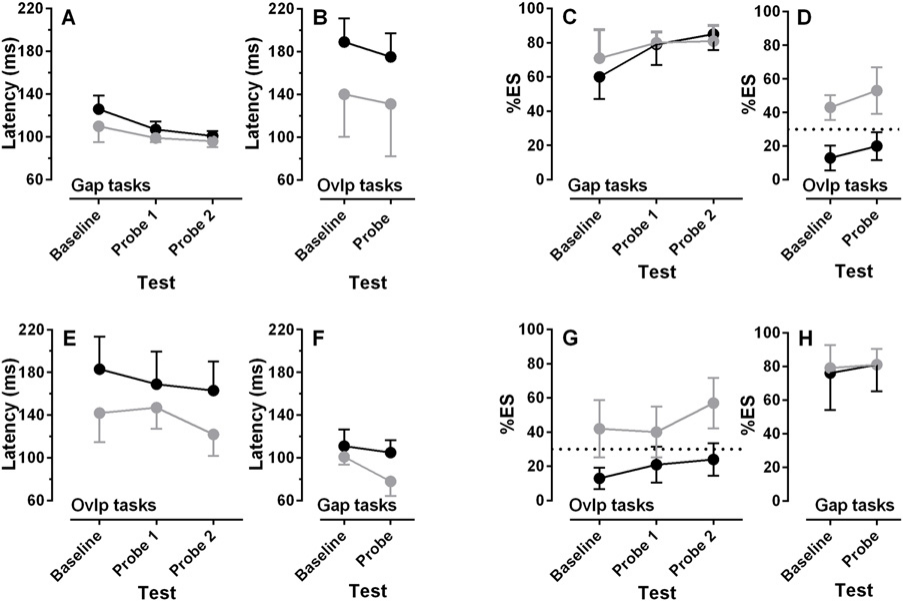
Effect of gap (A-D) and overlap (E-F) training on group data. Intersubject mean±95%CI saccade median latency (A,B,E,F) and intersubject mean±95% CI %ES (C,D,G,H) for ESM (●) and Norm (●) groups is shown. To improve clarity, error bars are only plotted in one direction where they would extensively overlap.

Gap training (Fig. 6A-E; Fig. 7A-D) reduced the median saccade latency and increased %ES in gap conditions (Fig. 6A-C). For ESMs the intersubject mean (±SD) median latency was reduced from a baseline value of 110±16ms to 99±4ms in the first gap probe task, while the %ES increased from 71±18% to 80±7%. Larger effects were observed in the Norm participants; the comparable figures were a latency decrease from 126±14ms to 107±8ms and increase in %ES from 60±14% to 79±13% respectively. Lower median latencies and a higher %ES were observed in the second set of gap probe data, collected on day 12, after the participants had a break from testing (ESM latency 96±6ms, %ES 81±10%; Norm latency 101±5ms, %ES 85±10%; Fig. 7A,C). Thus for ESMs, between baseline and the second probe task there was a total decrease of 6ms in latency, and total increase of 10% in the %ES, while for the Norms latency decreased by 25ms, and the %ES increased 19%.

Comparison of overlap performance at baseline and after gap training (Fig. 6D,E; Fig. 7B,D) suggested that effects were less marked and more variable than was observed for gap performance (compare the change between Fig. 6A and B with that between D and E). There were decreases in median latency (Fig. 7B) and increases in %ES (Fig. 7D) which were similar in both groups. Comparing baseline and probe overlap performance, latency decreased from 140±43ms to 131±53ms while %ES increased from 43±18% to 53±15% in the ESMs. In the Norms latency decreased from 189±24ms to 175±24ms, while %ES increased from 13±8% to 20±9%. Thus in the Norm group, in which large changes were observed in gap tasks with gap training, much smaller changes were observed in overlap performance after gap training.

As the critical issue is whether it is possible to “train” non-ESM participants to behave like ESMs, we subjected the %ES and latency data for overlap tasks performed before and after gap training to similar 2x2 repeated measures ANOVAs. Group (ESM vs Norm) was treated as a between and session (baseline vs probe) as a within subjects factor. %ES was significantly higher in the probe compared to the baseline session (baseline = 28.4±20.7%, probe = 36.4±20.6%; F_1,12_ = 6.9, p = 0.02), with the same pattern holding for the latency data (intersubject mean of medians: baseline = 164±37 ms, probe = 153±31 ms; F_1,12_ = 7.6, p = 0.017). Importantly, group also returned a significant result for both %ES (ESM = 48±16.9%, Norm = 16.8±8.7%; F_1,12_ = 23.8, p<0.001) and latency (ESM = 135±25 ms, Norm = 182±25 ms; F_1,12_ = 13.8, p = 0.003). There was no interaction between the two factors for either %ES or latency data.

### Experiment 2: Effects of overlap training

In general the effect of overlap training (Fig. 6F-J; Fig. 7E-H) was proportionately less marked and more variable than the effects of gap training. In absolute terms, in overlap tasks latency declined from 142±26ms at baseline, to 122±19ms in probe 2, and %ES increased from 42±16% to 57±14% in ESMs. The comparable figures for the Norm group were a latency decrease from 183±29ms to 164±26ms, and an increase in %ES from 13±6% to 24±9%. For gap tasks before and after overlap training, there was a marked decrease in latency in the ESMs (from 101±7%ms to 78±13%ms), with a smaller decrease in the Norm group (from 111±15ms to 105±11ms; Fig. 7F). However, these changes were not driven by changes in the %ES (Fig. 7H).

As the effect of overlap training has not been reported previously, we analysed the main training data for both %ES and latency with 2x3 repeated measures ANOVAs with group (ESM vs Norm) as a between and session (baseline vs probe1 vs probe 2) as a within subjects factor. For %ES we found a significant effect of session (F_2,18_ = 12.3, p<0.001). Bonferroni post hoc comparisons indicated that significantly more ES were executed in probe 1 compared to baseline (p = 0.004) and in probe 2 compared to baseline (p = 0.016; baseline = 27.3±19.1%, probe 1 = 29.6±14.5%, probe 2 = 40.4±20.6%). Session also had a significant effect on latency (F_2,18_ = 7.5, p = 0.004); Bonferroni post hoc comparisons indicated that median latencies were significantly lower in Probe 1 compared to Baseline and in Probe 2 compared to Baseline (both p = 0.029; Baseline = 162±34.1 ms, Probe1 = 159±25.7 ms, Probe 2 = 143±30.8 ms). Group was significant, with ESMs exhibiting a higher %ES than Norms, (ESM = 46±15.5%, Norm = 19±9%; F_1,9_ = 22.2, p = 0.001) and saccades with lower latency than the Norms (ESM = 136±22 ms, Norms = 172±28 ms; F_1,9_ = 5.5, p = 0.04). These two factors did not interact with either %ES or median latency.

### Experiment 2: Effects of gap training on overlap latency distributions

**Fig 8:**
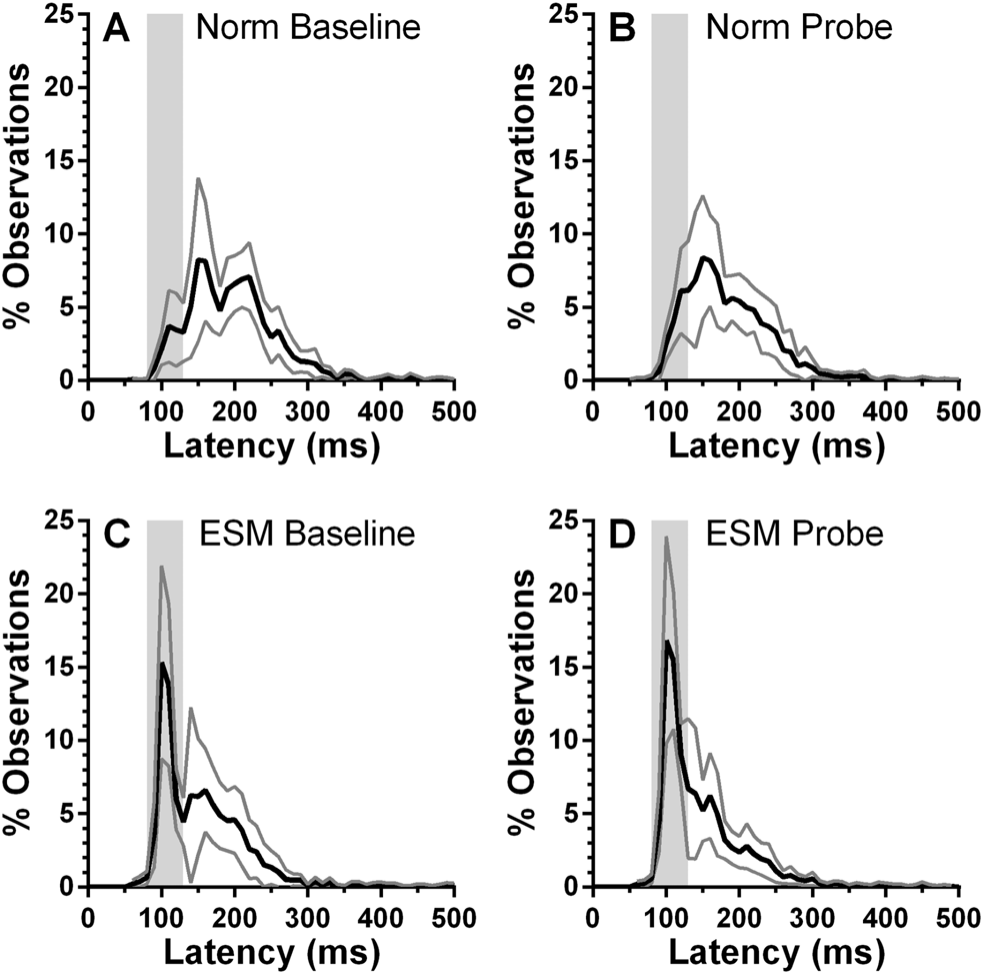
Effect of gap training on overlap latency distributions. A,B: average distributions for the Norm group. C,D: Distributions from the ESM group. Solid black line shows the mean, grey line the 95% CI. Grey vertical region illustrates the ES range.

We further examined the effect of gap training on overlap performance by constructing average distributions from the overlap tasks executed before and after gap training (Fig. 8). Smaller participant groups necessarily meant noisier distributions. While, as described above, the %ES in the Norm group clearly increased as a result of gap training, what we did not observe is the emergence of the type of early peak centred on 100ms that was clearly present in the ESMs. Post-training distributions (Fig. 8B,D) were similar to the average distributions computed for the two large groups of participants at the outset of testing (Fig. 2C,D).

## Discussion

Express saccade makers (ESMs), are healthy, adult, participants who execute high numbers of low latency (80 ms-130 ms) express saccades (ES) even in the overlap condition. As can be seen from Fig. 2, ESM saccade latency distributions for simple prosaccade overlap tasks (in which the fixation target remains illuminated when the saccade target appears), are marked by a large distinctive peak early in the distribution centred on 100ms (see [12] Fig. 2 and [13] Figure S1 for further examples of individual ESM latency distributions). The prominent ES peak is also a clear feature of average latency distributions (Fig. 2D). These have the same shape as the more commonly plotted pooled latency distributions, but have the advantage that the underlying intersubject variability is captured by means of calculating and plotting an appropriate measure of that variability (here the 95% CI). The narrow confidence interval around the ES latency peak, and the clear difference in this part of the distribution between ESM and non-ESM (“Norm”) participant groups, provides some reassurance that the individual example distributions are typical. Note that this pattern of behaviour persists across experimental setups and paradigms[15,23]. While this performance is striking, what was unclear until now is the extent to which it is temporally stable and how it is affected by training.

As the percentage of ES (%ES) in overlap conditions is the basis on which we have identified ESMs, it is the stability of this parameter both within and between sessions that we centred our analysis on. Analysis of data from large groups of ESM and Norm participants (59 and 54 participants respectively) suggested small differences in the %ES generated in two blocks of 200 trials in a single session, with a high intraclass correlation coefficient (ICC = 0.97, *p*<0.001) between the performance in the two blocks. Klein and Fischer (2005)[17] performed both split-half and odd-even analysis in 327 healthy participants for both the %ES and mean saccade latency and reported similarly high Pearson correlation coefficients (eg for their odd-even analysis—latency:0.97; %ES:0.96). They did not report whether any of their participants were ESMs.

There was no evidence in these data that there was any difference between our participant groups other than that for ESMs the %ES was obviously higher than for the Norm group. The regression line plotted in Fig. 3B calculated from the whole dataset appears to fit the ESM and Norm data equally well. When we calculated two separate regression lines, the slopes were statistically indistinguishable (F_1,109_ = 3.2; p = 0.08).

A similar pattern was observed when we examined test-retest reliability between sessions for relatively large participant groups (28 ESMs, 32 Norms), by comparing %ES recorded in two sessions an average of 87 days apart. However, we were also able to examine stability over three sessions in 30 participants (13 ESMs, 17 Norms; Fig. 4) with an average of 178 days (i.e. just over six months) between the first and last session. These data in particular suggest relatively stable performance over the three sessions, and a stable difference between the groups (as evidenced by the ANOVA reported in the results), with a high ICC (0.97). The test-retest reliability of saccade parameter measurements has been reported previously to decline over extended periods of up to 24 months [17,24]. For saccade latency Klein and Fischer (2005)[17] reported a correlation coefficient of 0.74 in 117 participants for latency and only 0.46 for %ES for two sessions approximately 19 months apart. However, the mean age of their participants was 12.2y (range 6–18y); the inclusion of relatively young participants whose oculomotor system was still maturing would be expected to contribute a degree of instability. Over 24 months Iancono and Lykken (1981)[24] reported a correlation of 0.54 for latency measured in 52 adult participants. Our results, albeit over a shorter timeframe, suggest stable performance overall, with no difference in stability of %ES between our two participant groups. In particular, a high %ES in the first session, was followed by a high %ES across sessions.

We addressed the issue of how well our original classification of participants operated over time. Clearly, as identified in the results, for a large majority of participants (93%) the classification obtained on the basis of their performance in 400 trials in the first session, fitted with subsequent performance over the six month period. We identified two participants who were marginally below the 30% criterion on that first session, but clearly above it on at least one subsequent session. However, as Fig. 4 illustrates, the 30% criterion level for defining ESMs, while in one sense arbitrary, does appear to capture something that another metric (eg median latency) might miss. This criterion originated in the early reports of ESMs [8], and was designed to capture participants responding in what was, at the time, an unexpected manner. It appears to do this reasonably well and consistently.

More broadly, an examination of the average latency distributions generated from these data (Fig. 5) also suggested consistent performance within groups and consistent differences between groups. There has been considerable interest in manual reaction time distributions and how they might reflect individual differences in underlying cognitive function [25], and also considerable effort in developing analysis approaches to oculomotor reaction times [26]. We have not conducted these more sophisticated analyses here because the average distribution histograms are sufficient to demonstrate two important features. Firstly, the prominent express peak has both a consistent position (i.e. is centred on the same latency) and magnitude in the ESM group across the three sessions and is absent in the Norm group. This necessarily means that that most prominent peak which is consistently observed in the Norm distributions (the “fast regular peak”) is greatly reduced across all three sessions in the ESMs. Secondly, that these differences are indicative of robust differences between groups is confirmed by the ANOVA that was undertaken. This demonstrated consistent differences between groups, but no differences between sessions. Overall we conclude that the criterion we have used to define our participant groups does classify them effectively, and that, thus classified there is good general stability in the %ES within groups at least over the timespan we have investigated.

What then of our attempts to alter the %ES using training? We confirmed that exposing participants to blocks of gap trials reduces median saccade latency and increases the %ES. Because the ESMs already produced high proportions of ES in gap conditions, larger proportionate differences were observed in the Norm group. For the Norm group, the alterations in both latency and %ES were very similar to those reported previously. Bibi and Edelman (2009[5]) using 6–12 sessions of gap training in nine participants observed a decrease of around 20ms in median latency, and an increase in %ES from 33% to 70%. Note that they used a different latency range to define ES (75 ms-110 ms as opposed to our 80 ms-130 ms). In an earlier experiment in which target direction was not randomised, smaller changes were observed with training, but in the same direction as those reported here[18]. We extended these earlier results by also investigating the effect of gap training on overlap performance, and found that there was much less of an effect on both median latency and %ES (see Fig. 6DE). While on average %ES did increase in overlap conditions with gap training, two observations should be noted. First, it increased by approximately the same in both groups, showing no evidence of a ceiling effect in the ESMs. Secondly, the increase in the Norms was not sufficient to raise the group average above the 30% criterion. Indeed the intersubject mean plus the upper 95% confidence limit only reached 29%. Overlap training produced a similar pattern of effects, although they tended to be smaller and more variable (see Fig. 6F-H). There was an increase in the %ES for both groups which was similar to the increase observed with gap training (compare Fig. 7D and G).

Given that training did have some effect on ES production in overlap conditions (albeit broadly equivalent in both groups and not large enough to change the classification of the groups) did this provide evidence that the Norm group became more “ESM-like” in their behaviour? Evidence that counts against this interpretation is provided by the average distributions for the overlap probe tasks from before and after gap training (Fig. 8). While in the Norm group there are clearly more saccades with latency in the express range, there is no evidence of the express peak that is observable in the ESM distributions before and after training. Gap training has been argued to better enable non-ESM participants to exploit stimulus features such as the warning signal supplied fixation offset to increase the efficiency of their response to target onset [5,18]. This, allied to the underlying effects of the absence of a foveal stimulus [2], is sufficient to explain a general reduction in latency, but apparently not the generation of ES and their overproduction in ESMs. However, given that overlap training clearly did reduce latency and increase ES production to some extent, the importance of gap-related stimulus features such as the fixation-offset effect should not be overestimated.

These training effects were produced by exposing participants to 400 trials per day for five consecutive days. Clearly, identical stimulus conditions to those we have employed do not occur in natural circumstances although it is conceivable that some specific environments (eg computer games) might replicate some features of the training regimes. This general absence of environmental stimuli that might increase ES production is also likely to be particularly the case for the gap stimuli that produced the largest and most consistent training effects. Currently fixated objects tend not to simply disappear. Therefore it seems unlikely that exposure to some environmental stimulus can account for the development of the pattern of saccade behaviour observed in the ESMs. Even if some feature of the visual environment were to “train” the overproduction of ES in the equivalent of overlap conditions, then presumably this would continue until each participant had reached some kind of natural limit. Yet we found that the %ES could still be driven higher in both participant groups by training, and by the same extent in overlap conditions.

We have confirmed that the production of ES is generally stable over extended periods of time, and that the overproduction of ES observed in ESMs in overlap conditions is a temporally stable feature of their performance. We have shown that intensive training is not sufficient to transform Norms into ESMs and that some particular feature of the visual environment is unlikely to produce the signature behavioural features that we have used to identify ESMs (the overproduction of ES in overlap conditions, forming a distinct early peak in the saccade latency distribution). We suggest that the pattern of behaviour of ESMs constitutes a particular and stable oculomotor phenotype, and provides an additional means of investigating relationships between saccade behaviour and specific aspects of cognitive function on the one hand, and the underlying neurogenetics of the oculomotor system on the other.
